# Insights into the structure, function, and impact of *Candida albicans UPC2* gene on azole resistance; a mini-review

**DOI:** 10.22034/cmm.2024.345248.1595

**Published:** 2024-12-31

**Authors:** Akbar Hoseinnejad, Amir Hossein Mahdizade, Maryam Erfaninejad, Firoozeh Kermani, Mona Ghazanfari, Aylar Arbabi, Seyed Sobhan Bahreiny, Arezoo Bozorgomid, Mojtaba Moradi, Iman Haghani, Mahdi Abastabar

**Affiliations:** 1 Department of Medical Mycology, School of Medicine, Ahvaz Jundishapur University of Medical Sciences, Ahvaz, Iran; 2 Student Research Committee, Ahvaz Jundishapur University of Medical Sciences, Ahvaz, Iran; 3 Department of Medical Genetics, Faculty of Medicine, Ahvaz Jundishapur University of Medical Sciences, Ahvaz, Iran; 4 Department of Basic Medical Sciences, Shoushtar Faculty of Medical Sciences, Shoushtar, Iran; 5 Infectious Diseases and Tropical Medicine Research Center, Health Research Institute, Babol University of Medical Sciences, Babol, Iran; 6 Invasive Fungi Research Center, Communicable Diseases Institute, Mazandaran University of Medical Sciences, Sari, Iran; 7 Department of Medical Mycology, Faculty of Medicine, Mazandaran University of Medical Sciences, Sari, Iran; 8 Physiology Department, School of Medicine, Tehran University of Medical Sciences, Tehran, Iran; 9 Medical Biology Research Center, Health Technology Institute, Kermanshah University of Medical Sciences, Kermanshah, Iran; 10 Department of Physiology, School of Medicine, Kermanshah University of Medical Sciences, Kermanshah, Iran

**Keywords:** Antifungal therapy, Azole resistance, *Candida albicans*, UPC2

## Abstract

**Background and Purpose:**

Candidiasis is a prevalent fungal infection caused by various species of *Candida*, especially, *C. albicans*. The emergence of resistance to azole medications, which are frequently prescribed for the treatment of *Candida* infections, presents a significant challenge in the management of these infections.

**Materials and Methods:**

The present mini-review summarizes findings from a comprehensive search of articles published between 1999 and 2024, retrieved from Scopus, PubMed, and Web of Science. Studies were selected using specific keywords based on relevance to *UPC2* gene functions, azole resistance mechanisms, and *C. albicans* biology.

**Results:**

The *UPC2* gene has become crucial in regulating drug resistance in *C. albicans*. This gene encodes a zinc (II)-Cys (6) transcription factor involved in the biosynthesis of sterols and contributes to resistance against azole antifungal drugs. When exposed to azoles, *UPC2* in *C. albicans* enhances the expression of ergosterol biosynthesis genes, such as *ERG2* and *ERG11*. Increased expression of *ERG11* leads to reduced susceptibility to azoles by boosting the production of 14α-lanosterol demethylase, the primary target of these antifungal agents. Furthermore, *UPC2* regulates sterol uptake under anaerobic conditions and manages other adaptations to environmental changes, all of which contribute to azole resistance.

**Conclusion:**

Gaining insight into how the *UPC2* gene contributes to azole resistance is essential for the development of effective strategies in the antifungal drug development process.

## Introduction

In recent decades, invasive fungal infections have increased significantly, reaching an unacceptable global annual incidence rate of 6.5 million cases with 3.8 million deaths, ~66% (2.5 million deaths) of which are directly attributable to these infections [ 1 ]. Invasive candidiasis is a serious infection caused by various species of *Candida*. It is the most common fungal disease found in hospitals in high-income countries. Global prevalence of this infection ranges from 250,000 to approximately 700,000 cases per year, with an incidence rate of 2-14 cases per 100,000 individuals. The mortality rates associated with this disease are 40-55% [ 2 ]. *Candida* infections are among the top 10 pathogens commonly identified in intensive care units or immunocompromised patients. They account for as much as 10% of bloodstream infections acquired in hospitals, outpacing several prevalent bacterial species, including *Pseudomonas aeruginosa* [ 3 ]. However, *Candida* species can also cause superficial infections [ 4 ]. Although *Candidaalbicans* remains the most commonly encountered pathogen in clinical samples, an epidemiological shift towards non-*albicans Candida* species has been repeatedly reported in many geographic areas, which is of growing concern worldwide [ 3 , 5 - 7 ].

The growing public health threat posed by new and old *Candida* species, and other fungal agents, has also been recognized by the World Health Organization, which recently published the first fungal priority pathogens list to raise awareness of fungal infections and support research on antifungal resistance [ 8 ]. This list includes 19 pathogens, most of which are intrinsically resistant to commonly used antifungal agents or capable of rapidly developing resistance upon repeated exposure to the drug [ 9 ].

**Figure 1:**
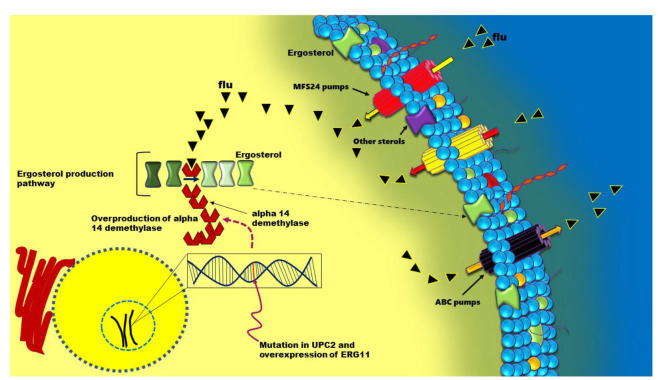
Graphical representation summarizing the reduced susceptibility to azoles of *Candida* spp. due to DNA changes occurring in the *UPC2* gene. Specific mutations of this gene result in the transcriptional upregulation of the *ERG11* gene leading to overproduction of the enzyme lanosterol 14-α-demethylase and, ultimately, to fluconazole resistance.

Azoles, echinocandins, and polyenes are classes of antifungal drugs commonly used to treat *Candida* infections, with azoles being the most frequently used for treatment and prevention [ 10 ]. Unfortunately, in recent years, the overuse of azoles for long-term prophylaxis or therapy has led to azole resistance as a growing problem, thus increasing the difficulty of treatment [ 11 , 12 ]. *Candida albicans* holds key genes and molecular mechanisms related to azole resistance [ 11 ], including the increased expression of drug efflux pumps that are encoded by the *CDR1*, *CDR2*, and *MDR1* genes, altered expression or mutations in the *ERG11* gene encoding lanosterol 14-α-demethylase, loss of heterozygosity at certain loci, particularly TAC1 and MRR1, and mutations in zinc cluster transcription factors, such as TAC1, MRR1, and UPC2, which work independently or together to create varying degrees of azole resistance in *C. albicans* (Figure 1) [ 11 , 13 ]. The UPC2 is a zinc (II)-Cys (6) transcription factor that plays a role in sterol biosynthesis and azole resistance. Initially recognized as a homolog of the *S. cerevisiae* (*ScUPC2*) gene, the *UPC2* gene in *C. albicans* has been shown to enhance the expression of ergosterol biosynthesis genes *ERG2* and *ERG11* when exposed to fluconazole [ 11 ]. Overexpression of *ERG11* has been shown to reduce azole susceptibility by increasing the production of the azole target 14α-lanosterol demethylase. Additionally, UPC2 controls sterol uptake without oxygen and regulates other environmental adaptations, including anaerobic conditions, contributing to azole resistance [ 14 ]. This review will explore the structure and function of the *UPC2* gene, as well as its involvement in azole resistance, addressing the existing knowledge gaps in this area.

### Data collection

The current mini-review was conducted through online literature searches of peer-reviewed articles published between January 1, 1999, and April 21, 2024, and indexed in the following three authoritative databases: Scopus, PubMed, and Web of Science. The inclusion and exclusion criteria in this study were as follows:

Inclusion criteria:

Studies on *C. albicans*: Articles focusing on the biology, genetics, and drug resistance mechanisms of *C. albicans*, specifically concerning the *UPC2* gene.

Azole resistance studies: Research that investigates resistance mechanisms to azole antifungal drugs, including mutations, regulatory pathways, or gene expression analysis.

Peer-reviewed publications: Articles published in peer-reviewed journals to ensure the reliability and scientific rigor of the data.

English language: Studies published in English to maintain consistency in interpretation.

Time frame: Articles published between January 1, 1999, and April 21, 2024, as defined in the manuscript.

Keywords: Studies using terms, such as “*UPC2* gene”, “*C. albicans*”, “azole resistance”, and “resistance mechanism.”

Exclusion criteria:

Non-English publications: Studies published in languages other than English.

Non-peer-reviewed sources: Articles from non-peer-reviewed sources, including preprints or gray literature.

Non-*Candida* species studies: Research focusing on organisms other than *C. albicans* (e.g., *Aspergillus* or non-*albicans Candida*).

Unrelated topics: Studies that did not address the *UPC2* gene, azole resistance, or related genetic and molecular mechanisms.

Inadequate data: Articles lacking sufficient experimental data or those that are purely theoretical without substantive evidence supporting their conclusions.

### Structure of UPC2 gene

According to the Candida genome database (www.Candidagenome.org), in *C. albicans*, the 2,139 bp long-*UPC2* gene (orf19.391) is located on chromosome 1 and encodes a zinc-cluster transcription factor of 712 amino acids. The gene has orthologues in other pathogenic *Candida* species (*C. auris*, *C. dubliniensis*, and *C. parapsilosis*) and other fungi, including Saccharomyces cerevisiae where an additional paralogous gene (*ECM22*) is also present (www.yeastgenome.org) [ 15 - 17 ].

**Figure 2:**
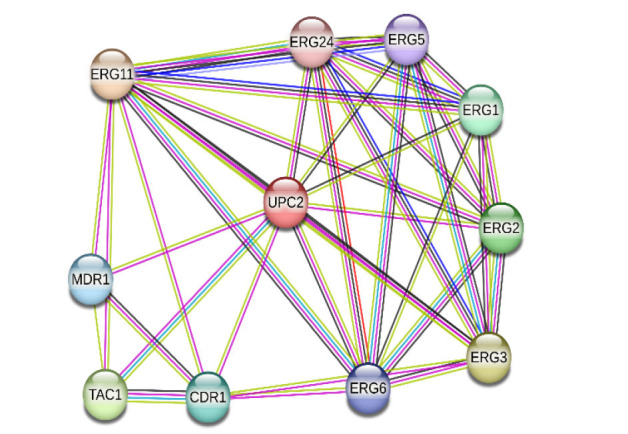
A protein-protein communication network between *UPC2* and other effector gene products in *C. albicans*. The interaction network was constructed using the STRING database version 11.0 (https://string-db.org). The confidence score threshold was set to be more than 0.7, while the species was "*C. albicans*" (Cytoscape version 3.7.1, www.cytoscape.org). The edges are categorized based on the source of interaction data: known interactions are derived from curated databases (represented by blue lines) and experimentally determined interactions (pink lines), while predicted interactions are indicated by green lines (gene neighborhood), red lines (gene fusions), and blue lines (gene co-occurrence). Additionally, other sources of associations include text mining (yellow lines), co-expression (black lines), and protein homology (light blue lines).

This family of transcriptional regulators, with over 80 members, is a highly conserved fungal-specific family of transcription factors with a Zn(2)-Cys(6) binuclear cluster domain [ 18 ].Sequence analysis of the *UPC2* gene in *C. albicans* has revealed two key domains, namely an anchoring transmembrane domain and a region associated with transcription factors that include several nuclear localization signals and a fungal Zn(2)-Cys(6) binuclear cluster domain [ 18 ]. The UPC2 regulates the expression of *ERG11* and most of the genes in the ergosterol biosynthesis pathway on some level (Figure 2) [ 19 ]. Ergosterol is a fundamental component of the fungal cell membrane, and, therefore, its biosynthetic pathway is the primary target for most antifungal compounds currently used to treat severe fungal infections (allylamines, azoles, and morpholines) [ 20 ].

Azole exposure can lead to overexpression of the *UPC2* gene, which can adequately compensate for the inhibition of target enzymes [ 19 ]. While UPC2 disruption results in reduced accumulation of exogenous sterols and hyper-susceptibility to fluconazole [ 21 ]. Previous studies have indicated that UPC2 may also trigger MDR1 expression, playing a minor role in the regulation of multidrug efflux pumps and MDR1-mediated drug resistance. However, MRR1 is a more significant regulator of this efflux pump [ 22 ].

**Figure 3:**
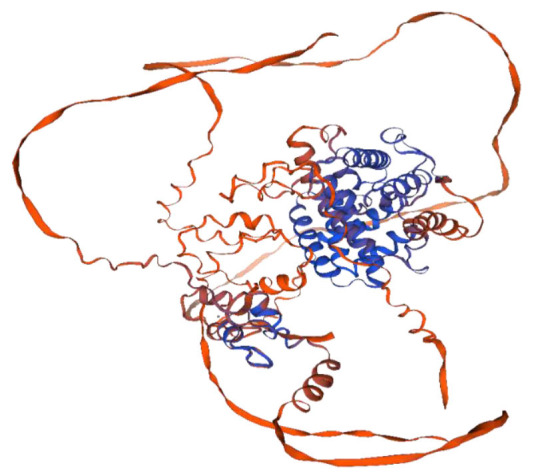
A 3D homology-based model of UPC2 protein predicted using the Swiss Model tool on the ExPASy server. The ribbon model shows the protein backbone with a color gradient from blue to orange and red. The blue regions represent the structured core or DNA-binding domain that is essential for protein function, while the orange to red regions represent more flexible regions that may facilitate interactions with other molecules or allow the protein to change its shape according to functional-regulatory needs. Amino acids at positions 54-81 (Sequence: CSTCKKRRVKCDEQRPVCGNCTKLKLDC) are the DNA binding region.

Zinc cluster family of transcription factors, which includes TAC1p, MRR1p, and UPC2p proteins, is exclusive to fungi. These transcription factors are crucial for helping *C. albicans* cells to adapt to drug pressure and other general stresses [ 23 , 24 ]. The *UPC2* gene in *C. albicans* encodes a *712 amino acid protein* essential in sterol biosynthesis regulation and drug resistance [ 25 ]. The structure of the UPC2 protein (Figure 3) includes different domains with specific structural and functional features, essential for successful interaction with cellular targets. For example, the amino-terminal (N-terminal) DNA-binding domain, characterized by a conserved Zn (II)2-Cys6 zinc finger motif, is essential for the response to sterol levels in the cell [ 17 , 26 ]. While the carboxyl-terminal (C-terminal) regulatory domain is fundamental for responding to intracellular sterol levels and their regulation [ 27 , 28 ]. In addition, the UPC2 protein also contains a C-terminal ligand-binding domain (LBD) consisting of 11 α-helices and connecting loops, folded to form a closed clamp that creates a deep hydrophobic pocket in the core of the protein, providing enough space for binding sterol molecules [ 28 ]. The C-terminal LBD of UPC2 adopts a novel α-helical fold involved in ligand binding, particularly sterols, like ergosterol [ 28 ].

Gain-of-function (GOF) mutations in UPC2 can lead to increased expression of the *ERG11* gene encoding for the lanosterol 14α-demethylase, an essential enzyme of the ergosterol biosynthetic pathway [ 19 , 29 ]. This protein is the target of the azole drugs and its inhibition results in a depletion of ergosterol, accumulation of toxic sterols, and inhibition of fungal growth [ 30 , 31 ]. Overexpression of the *ERG11* gene has been found to lead to higher levels of cellular ergosterol and reduced susceptibility to azole drugs by enhancing the production of the enzyme that targets azoles [ 11 ].

Sequence analysis of UPC2 alleles of fluconazole-resistant isolates showed that specific single-nucleotide polymorphisms (SNPs) generate dominant GOF mutations, such as the *UPC2G648D*, *UPC2Y642F* or *UPC2A643V* amino acid substitutions. This leads to a constant upregulation of the *ERG11* promoter, which in turn, grants fluconazole resistance to clinical isolates of *C. albicans* [ 29 ]. However, the exact mechanism underlying *UPC2*-mediated gene upregulation still remains unknown [ 29 , 32 ]. Types of *mutations* of the *UPC2* gene *investigated in* previous studies are listed in Table 1.

**Table 1:** Overview of studies investigating different types of mutations in the *UPC2* gene.

Author (year) Reference	Strains	Mutation of UPC2	Function
Nico Dunkel (2008) [ 21 ]	S2 (Fluconazole-resistant isolate from the patient)- *Candida albicans*	G648D	Elevated levels of *ERG11* expression and imparted fluconazole resistance
Clemens J. Heilmann (2009) [ 32 ]	Isolate 5052 (Fluconazole-resistant isolate from the patient)- *C. albicans*	G1927A	This resulted in an exchange of alanine for threonine at position 643 (A643T) in the encoded protein and increased *ERG11* and *UPC2* expression
Florent Morio (2013) [ 39 ]	CAAL28: HIV^1^ patient (*C. albicans*)	I142S^h3^, A451V^h^	Not mentioned
	CAAL37: Rheumatoid arthritis patient (*C. albicans*)	R68K^h^, I142S^h^, S190N^h^, S228N^h^	Not mentioned
	CAAL 61: HIV patient (*C. albicans*)	I142S, G648S	GOF mutation
	CAAL 67: HIV patient (*C. albicans*)	I142S^h^	Not mentioned
	CAAL74: SCID^2^ patient (*C. albicans*)	R68K^h^, I142S, S228N^h^, T273A^h^, G648S^h^, K684E^h^	GOF mutation, T273A and K684E: Elevated levels of *ERG11* expression and imparts fluconazole resistance
	CAAL 75: HIV patient (*C. albicans*)	R68Kh, I142Sh, S228Nh, K684Eh, T273Ah	T273A and K684E: Elevated levels of *ERG11* expression and imparts fluconazole resistance
Stephanie *A. Flowers* (2012) [ 29 ]	6 fluconazole-resistant clinical *ERG11*-overexpressing isolates	G648D	Elevated levels of *ERG11* expression and imparted fluconazole resistance
	9 fluconazole-resistant clinical ERG11-overexpressing isolates	G648S	Elevated levels of *ERG11* expression and imparted fluconazole resistance
	1 fluconazole-resistant clinical *ERG11*-overexpressing isolates	A643T	Elevated levels of *ERG11* expression and imparted fluconazole resistance
	2 fluconazole-resistant clinical *ERG11*-overexpressing isolates	A643V	Elevated levels of *ERG11* expression and imparted fluconazole resistance
	2 fluconazole-resistant clinical *ERG11*-overexpressing isolates	A646V	Elevated levels of *ERG11* expression and imparted fluconazole resistance
	2 fluconazole-resistant clinical *ERG11*-overexpressing isolates	Y642F	Elevated levels of *ERG11* expression and imparted fluconazole resistance
	3 fluconazole-resistant clinical *ERG11*-overexpressing isolates	W478C	Elevated levels of *ERG11* expression and imparted fluconazole resistance
	2 fluconazole-resistant clinical *ERG11*-overexpressing isolates	G304R	This mutation does not result in the gain of function
Samantha J. Hoot (2011) [ 36 ]	17 azole-resistant strains of *C. albicans*	A643V	In the A643V clinical isolates and reconstructed strains, there were increased levels of azole susceptibility, ergosterol, and expression of *ERG* genes
Christina Popp (2017) [ 37 ]	F5 (fluconazole-resistant clinical isolate, *C. albicans*)	G648S	*ERG11* overexpression and increased drug resistance
		G1942A	Not mentioned
	TW17 (fluconazole-resistant clinical isolate, *C. albicans*)	A643V	*ERG11* overexpression and increased drug resistance
Roy A. Khalaf (2021) [ 41 ]	CA77 (clinical isolate, *C. albicans*)	I142S	A recognized mutation that has not been previously associated with antifungal resistance
Emilie Sitterlé (2020) [ 40 ]	151 unrelated *C. albicans* strains susceptible to fluconazole and caspofungin	L25F^h^, K26E, T41N^h^, R68K, I142S, T144I^h^, S190N, A221P, S228N, N250S^h^, T273A, G321S^h^, L342V^h^, V507I^h^, L635W^h^	No conferred increases in azole MICs

1 : Human immunodeficiency virus;

2 : Severe combined immunodeficiency; h: heterozygous (mutation in a single allele);

MIC: minimum inhibitory concentration

## Discussion

The most frequently prescribed antifungal medications for infections caused by *C. albicans* are azoles, which work by inhibiting the enzyme 14-α-sterol demethylase, encoded by the *ERG11* gene [ 33 ]. Nucleotide changes in this gene can generate missense mutations affecting the azole-binding domain of the protein, thus conferring resistance or reduced susceptibility to these drugs. Moreover, overexpression of *ERG11* leads to the increased production of the target enzyme, contributing to the evolution of azole-resistant isolates. An important key regulator of the expression of the *ERG11* gene is the zinc cluster protein UPC2 whose inhibition leads to hypersensitivity to azoles and a reduced uptake of alternative sterols into *C. albicans* cell membrane. Recently, the critical role of UPC2 in *C. auris* was highlighted by the regulation of the activation of the MRR1/MDR1 pathway and the ergosterol biosynthesis pathway [ 34 , 35 ]. Exposure of *Candida* to azole leads to GOF and overexpression of *UPC2*, which inhibits the target enzymes and results in resistance to azoles [ 21 ]. McPherson et al. and Silver et al. introduced the UPC2p as a key regulator of ergosterol metabolism in *C. albicans* [ 17 , 18 ] who first demonstrated that in the absence of UPC2, the azole-inducible expression of *ERG2*, *ERG7*, *ERG11*, and *ERG25* genes was decreased.

Researchers have identified numerous substitutions in the UPC2 gene linked to the development of azole resistance. Previous studies have mainly focused on the mutation related to the I142S amino acid substitution in the UPC2 protein. However, it does not appear to be linked to azole resistance since it was also detected in azole-susceptible *C. albicans* isolates [ 25 ].

The A643V substitution in the *UPC2* is another mutation that increases fluconazole resistance in clinical *Candida* isolates [ 36 ]. This mutation affects the C-terminal regulatory domain of UPC2 disrupting its function and upregulating the *ERG11* expression in resistant isolates [ 36 ].

In a study conducted by Flower et al. in 2012 [ 29 ], eight distinct mutations of the *UPC2* gene (G648D, G648S, A643T, A643V, Y642F, G304R, A646V, and W478C) were identified in 29 out of 47 isolates of *C. albicans*. Notably, seven of these mutations (87.5%) led to higher expression levels of the *ERG11* gene and increased cellular ergosterol, resulting in decreased sensitivity to fluconazole when compared to wild-type strains. Genes affected by these variations were found to be involved in ergosterol biosynthesis, oxidoreductase activity, and the major efflux pump encoded by the MDR1 gene [ 29 ]. The UPC2 GOF mutations are common in clinical isolates, but not necessarily in all isolates less susceptible to azoles [ 37 ]. For example, the highest expression of the ERG11 gene is associated with the amino acid substitution G648D and, therefore, different effects might be observed in the mutant strains during antifungal susceptibility testing, depending on the type of substitution and the antifungal drug used [ 38 ]. Previous studies have shown that the strains expressing the G304R mutant allele did not have any decrease in antifungal susceptibility. In contrast, an increase in MIC values for fluconazole and terbinafine was observed in the strains expressing G648D, G648S, A643T, A643V, Y642F, W478C, and A646V mutations [ 29 ].

In another study, Popp et al. in 2017 [ 37 ] identified three UPC2 GOF mutations, namely G648S, G1942, and A643V, in two fluconazole-resistant clinical C. albicans isolates, F5 and TW17. They found these mutations caused a significant fitness defect when isolates were grown for 24 h in a yeast extract-peptone-dextrose medium. Furthermore, the same authors noted that the fitness costs of drug resistance are also likely to depend on the individual host [ 37 ].

In line with previous studies [ 39 , 40 ], Khalaf et al. [ 41 ] utilized whole genome sequencing to identify 19-point mutations across eight genes (*ERG11*, *ERG24*, *ERG251*, *UPC2*, *CDR1*, *MRR2|*, *FKS1*, and *GLS1*) in the azole-resistant *C. albicans* strain known as CA77, which was isolated from a stool sample. Other UPC2 mutations were identified by large-scale genome sequencing performed by Sitterlé et al. [ 40 ], and two substitutions, D654N and G657D, were reported as new putative mutations involved in antifungal resistance.

Given the increasing resistance of *Candida* species to common antifungal agents, especially azoles [ 42 ], and also considering the high plasticity of *Candida* genomes [ 43 , 44 ], it is important to identify rapid DNA mutations that lead to drug resistance under different environmental conditions. Based on our review of various studies from 1997 to 2024, most of the mutations identified in the *UPC2* gene so far result in overexpression of the *ERG11* gene, although the role of some of them is still unclear. 

## Conclusion

Overall, the *UPC2* gene plays a key role in the emergence of *Candida*-resistant phenotypes to azole antifungal drugs. With scientific advances in the use of new DNA sequencing techniques, different resistance pathways are expected to be discovered in the future. Knowledge of the molecular mechanisms underlying antifungal resistance and point mutations in genes involved in this phenomenon is important to guide the development of novel fungal therapeutics and diagnostics.
